# Research hotspots and frontiers in the tumor microenvironment of colorectal cancer: a bibliometric study from 2014 to 2024

**DOI:** 10.3389/fonc.2025.1525280

**Published:** 2025-02-05

**Authors:** Xinran He, Tingyi Xie, Li Shi, Xuyi Kuang, Lei Li, Xingyu Shang, Bo Fu

**Affiliations:** ^1^ The Fourth Clinical Medical College of Guangzhou University of Chinese Medicine, Shenzhen, Guangdong, China; ^2^ Department of Nephrology, Shenzhen Traditional Chinese Medicine Hospital, Shenzhen, China

**Keywords:** colorectal cancer, the tumor microenvironment, bibliometric analysis, hotspots, WoSCC

## Abstract

**Background:**

Colorectal cancer (CRC) is the second leading cause of cancer deaths globally, which poses a heavy burden on our healthcare and economy. In recent years, increasing researches suggest that the tumor microenvironment (TME) influences cancer onset, progression, metastasis, and treatment. This has become a popular direction for researching and attacking cancer. However, to date, there is no bibliometric analysis of colorectal cancer and tumor microenvironment from 2014 to 2024. This study aims to provide a comprehensive picture of the current research status, hotspots, and future trends in this field from a bibliometric perspective.

**Methods:**

In this study, the publications about colorectal cancer and tumor microenvironment from 2014 to 2024 were searched based on the Web of Science Core Collection database. Then we analyzed and visualized the data using CiteSpace, VOSviewer, bibliometrix package, and Microsoft Excel 2019.

**Results:**

A total of 748 publications were included in our study, and the number of publications entered a period of rapid growth after 2019. China and the United States are the major research and collaboration centers in this field. Elkord, Eyad is the most prolific author, and Frontiers in Immunology is the journal that published the most papers on the TME of CRC. In addition, keyword and cluster analysis showed that immune checkpoint inhibitors, cancer-associated fibroblasts, macrophage polarization, intestinal microbiota, colorectal cancer liver metastasis, drug resistance, scRNA-seq, etc. may be the research hotspots and trends in this field.

**Conclusions:**

Colorectal cancer and tumor microenvironment research is in the developmental stage, and strengthening international cooperation can help to drive this field forward. The main components and signaling in TME, CRC immunotherapy, colorectal cancer liver metastasis, and new research techniques are the hot research directions in this domain. Our findings will provide scholars with an up-to-date perspective on the current state of research, hotspots, and future trends in this field.

## 1 Introduction

According to the 2022 global cancer statistics published by the International Agency for Research on Cancer (IARC), colorectal cancer (CRC) ranks as the third most prevalent malignant tumor worldwide, with an incidence rate of approximately 9.6%. However, it possesses the second-highest mortality rate among all cancers, accounting for 9.3% of global cancer fatalities. Additionally, research has demonstrated that CRC patients are becoming younger, representing the primary cause of mortality among young men and the second-leading cause of death among young women in America, so imposing a significant burden on healthcare systems and the economy (1, 2). Currently, surgery, chemotherapy, and radiotherapy are still the main clinical interventions for CRC; nevertheless, the prognosis for advanced and metastatic CRC is unfavorable, and both chemotherapy and radiotherapy entail considerable adverse effects. In recent years, targeted therapies and immunotherapies have yielded substantial outcomes in CRC, with numerous investigational therapeutic strategies focusing on targeted therapies for driver gene mutations, such as EGFR, VEGF, BRAF, KRAS, MEK, as well as immune checkpoint inhibitors and their combinatorial approaches (3, 4). However, consequent gene mutations in different signaling pathways and immune escape lead to recurrence, metastasis, and drug resistance in CRC. Therefore, understanding the specific mechanisms of drug resistance and developing novel targeted or immunosuppressive drugs that overcome drug resistance and produce better efficacy and lower side effects are the key research directions for CRC in the future (5).

As our understanding of cancer continues to revolutionize, more and more evidence suggests that the complexity of the tumor microenvironment (TME) and its diversity across organs and patients promote cancer onset, progression, and metastasis, as well as mediate tumor cell drug resistance. Consequently, the TME is also gradually becoming an important direction for researching and attacking cancer. The TME is an intricate network of heterogeneous cell populations and non-cellular components, which mainly consist of immune cells, cancer-associated fibroblasts (CAFs), endothelial cells, extracellular matrix (ECM), and blood vessels. Among them, immune cells mainly include innate immune cells (tumor-associated macrophages (TAMs), tumor-associated neutrophils (TANs), tumor-infiltrating lymphocytes (TILs), and myeloid-derived suppressor cells (MDSCs), etc.) and adaptive immune cells (T cells and B cells). They are essential for tumor proliferation, tumor angiogenesis, immune escape, immunosuppression, and metastasis by secreting a variety of chemokines, cytokines, and growth factors while also interacting with tumor cells (6–8). As a result, it is necessary to clarify the TME of CRC to identify new core therapeutic strategies to overcome drug resistance and enhance patient prognosis.

Bibliometrics became an independent discipline in 1969 and has been widely used for literature analysis (9), which is an emerging method for evaluating the state of development and research hotspots within a particular field. Bibliometric analysis has been applied in various fields of medicine, providing comprehensive data regarding countries, institutions, authors, journals, keywords, and references, which can be used by researchers to discover new information about diseases, drug treatments, and broader trends in healthcare dynamics (10). Further, quantitative analysis of existing literature with the aid of modern computer technology and visualization tools such as CiteSpace, VOSviewer, and bibliometrix package can help to uncover the intrinsic links between information, for example, the collaborative relationships and research priorities of different countries, institutions, and authors. This can provide a more objective and comprehensive understanding of the development and the hot research trends of a particular field. At present, there is no bibliometric analysis of the TME and CRC. So the current status, hotspots, and frontiers of research in this domain are not yet clear. Thus, this study will systematically compile and analyze the literature on the TME and CRC published from 2014 to 2024, whereas it will comprehensively demonstrate the research status, hotspots, and future trends in this field through knowledge mapping.

## 2 Materials and methods

### 2.1 Data acquisition

**Figure 1:**
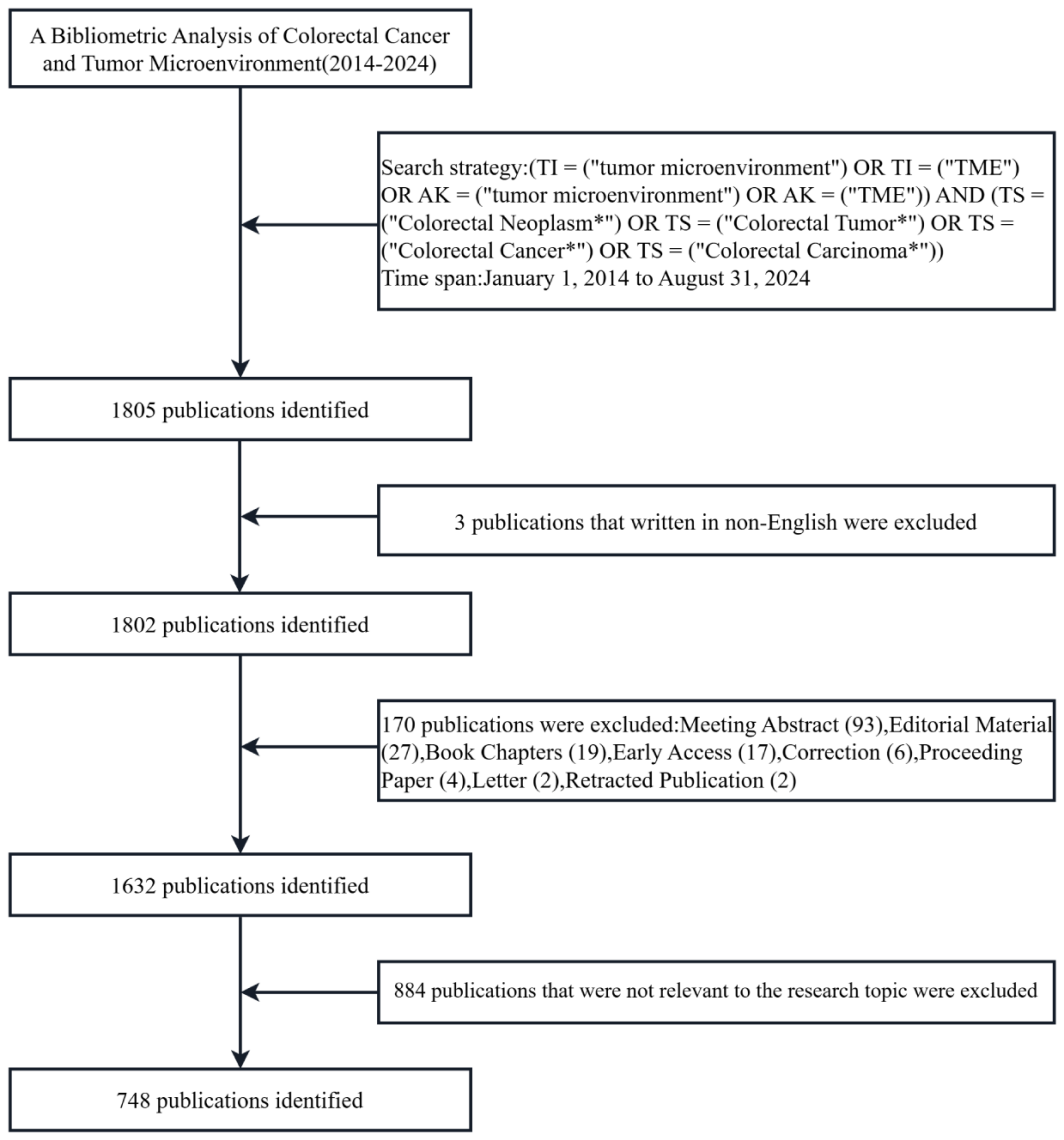
Flowchart for publications screening of the tumor microenvironment in colorectal cancer.

Data for this study were obtained from the Web of Science Core Collection (https://www.webofscience.com/wos/woscc/basic-search), which has been recognized by researchers all over the world as a high-quality digital literature resource database. Undoubtedly, it is regarded as the most appropriate database for bibliometric analysis. Meanwhile, to ensure the comprehensiveness and accuracy of the search results, the indexes were selected as Science Citation Index Expanded (SCIE) and Social Science Citation Index (SSCI) (11, 12). The search strategy for this study was (TI = (“tumor microenvironment”) OR AK = (“tumor microenvironment”) OR TI = (“TME”) OR AK = (“TME”)) AND (TS = (“Colorectal Neoplasm*”) OR TS = (“Colorectal Tumor*”) OR TS = (“Colorectal Cancer*”) OR TS = (“Colorectal Carcinoma*”)), with a search deadline of September 6, 2024. Further screening was conducted based on the following criteria: (1) the publication period was from January 1, 2014, to August 31, 2024; (2) the language was set to English only; (3) the type of publication was selected Article and Review while Meeting Abstract, Editorial Material, Book Chapters, Early Access, Correction, Proceeding Paper, Retracted Publication, and other literature types were excluded; (4) to ensure the quality and accuracy of the included publications, manual screening was performed by reading the abstracts and full texts to exclude publications that were not related to the TME in CRC. The entire screening procedure is illustrated in **Figure 1**. All data retrieval, screening, and extraction were carried out independently by two authors and cross-validated; any issues were resolved by a third author through negotiation. A total of 748 valid publications were included, which were exported as “plain text”, including “complete records and cited references”.

### 2.2 Research tools and visual analysis

This study used the bibliometrix package in R (version 4.3.3, https://www.bibliometrix.org), CiteSpace (6.1.R6 version), and VOSviewer (version 1.6.20) for bibliometric analysis, which have their strengths and complement each other. The Bibliometrix package in R provides a set of tools for quantitative bibliometrics and scientometrics research (13), utilized in this article for visualizing and analyzing national geographies, journal publication trends, and keywords. CiteSpace is a Java-based visualization and analysis tool developed by Prof. Chaomei Chen, which uses an incremental knowledge domain visualization approach to detect and visualize emerging trends and transient patterns in the scientific literature (14). We imported 748 publications into CiteSpace for de-duplication and the results showed no duplicate records. The period was set from January 2014 to August 2024, with a time slice of 1 year and a g-index of k=25. The institutional and author co-occurrence networks, journal dual map overlays, keyword co-occurrence networks, keyword timeline views, and citation bursts of references were visualized separately. The size of each node circle represents the number or frequency of occurrences, the color shade indicates the year of publication, and the connecting lines between nodes reflect collaborations. VOSviewer, on the other hand, is a software for presenting large bibliometric maps in an easy-to-interpret manner (15), providing visualization results of co-cited authors and co-cited references. Finally, Microsoft Excel 2019 was used for data statistics and building predictive models.

## 3 Results

### 3.1 Annual volume and trend of publications

**Figure 2:**
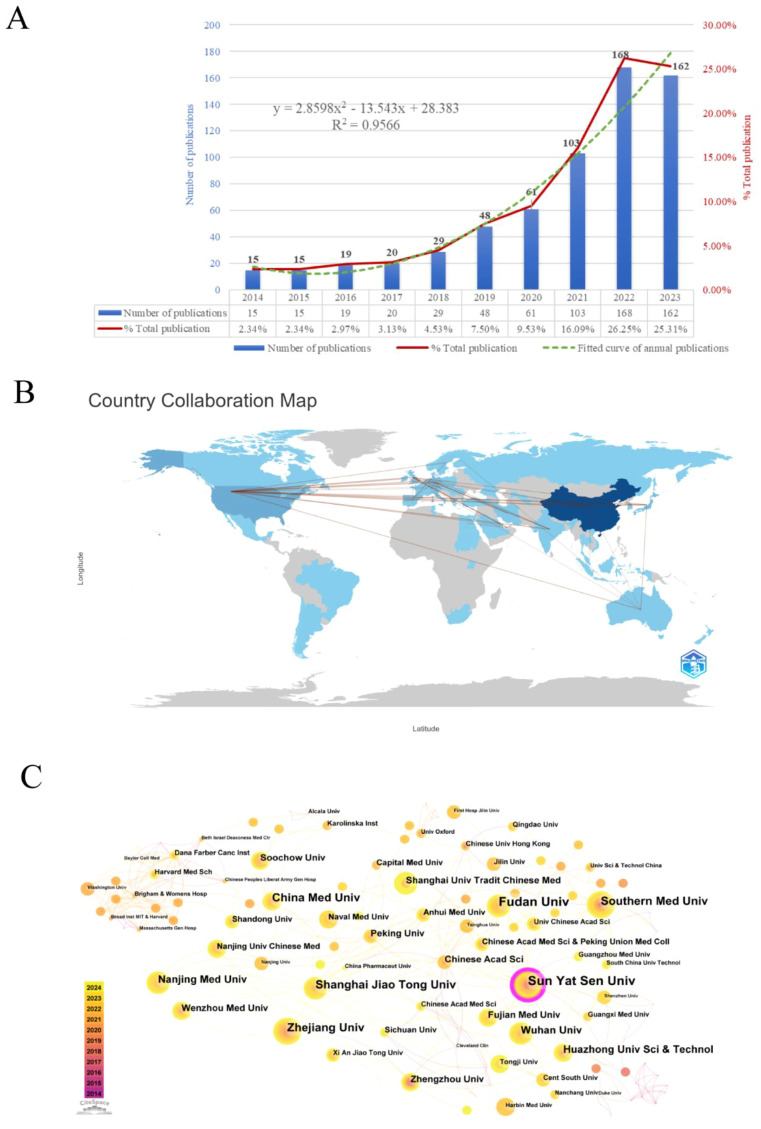
**(A)** Annual number of publications and trend chart. **(B)** Map of national/regional publications and collaborations. **(C)** Institutional co-occurrence and cooperation network map.

**Figure 2A** illustrates the annual volume and trend of publications related to the research area of the TME in CRC. In general, the quantity of publications in this domain keeps rising, with an annual growth rate of 23.01%. The period can be roughly categorized into two phases. The initial phase spanned from 2014 to 2018, during which the annual publication count stayed below 30. The second phase, post-2019, demonstrated a consistent increase in publishing volume, peaking at 168 publications in 2022, which constituted 26.25% of the total, and was 11 times the number of publications in 2014. This implies that the TME of CRC has garnered more and more attention from scholars in recent years, becoming a significant research hotspot in the field of oncology. As of the analysis in 2024, a cumulative total of 108 papers had been published. A binomial fitting model was constructed utilizing Microsoft Excel 2019: y = 2.8598x² - 13.543x + 28.383 (R² = 0.9566), with an anticipated total of approximately 179 publications by the end of 2024.

### 3.2 Analysis of national/regional cooperation

This study conducted a statistical analysis of publication counts from 59 countries/regions, with **Supplementary Table S1** enumerating the top 10 countries/regions in terms of the number of publications. The results reveal that China ranks upper with 394 publications, accounting for 52.7% of the total publications in this field, signifying a highly productive country in this domain, followed by the United States (66), Germany (37), and Japan (36). The terms single country publications (SCP) and multiple countries publications (MCP) respectively refer to joint publications from a single country and several countries. China has the highest number of articles co-authored with other countries, with 25, followed by the United States with 23. This underscores the close collaboration between China, the United States, and other countries. Secondly, China (6021) is the top-ranked country in terms of the total amount of literature citations, but its average number of citations per paper is low (15.3), which indicates that there is still a need to enhance the quality and academic impact of research. Moreover, France has the highest average number of citations per publication (74.8), with 11 papers receiving 823 citations, while the United Kingdom ranks second (51.4). The bibliometrix package is used to display the number of publications and collaborations in each country/region (**Figure 2B**). The color shades denote how many publications there are in these regions and the connecting lines represent the collaborations between them. As can be seen from the figure, the main collaborations occur between China and the United States, the United States and Japan, and the United States and the United Kingdom.

### 3.3 Analysis of institutions

Over the past decade, 1,167 institutions have participated in publishing papers relevant to the TME of CRC. The top 10 institutions with the highest outputs are listed in **Supplementary Table S2**, all of which are medical schools in China. Among them, Sun Yat-sen University (30) published the most research papers in this field, succeeded by Fudan University (26), Zhejiang University (21), Shanghai Jiao Tong University (20), and China Medical University (20). Besides, in order to thoroughly explore the collaborative relationships between these institutions, we used CiteSpace to visualize the institutional co-occurrence collaboration network, generating a total of 313 nodes and 487 edges, the number of which represents the close collaboration among mature institutions. As shown in **Figure 2C**, Sun Yat-sen University, with the highest centrality, is situated in the center and establishes strong collaborative relationships with Chinese medical institutions, including Shanghai Jiaotong University, Fudan University, and Zhejiang University. In addition, Harvard Medical School collaborates with Dana-Farber Cancer Institute, Brigham & Women’s Hospital, Washington University, and various other foreign institutions.

### 3.4 Analysis of journals

**Figure 3:**
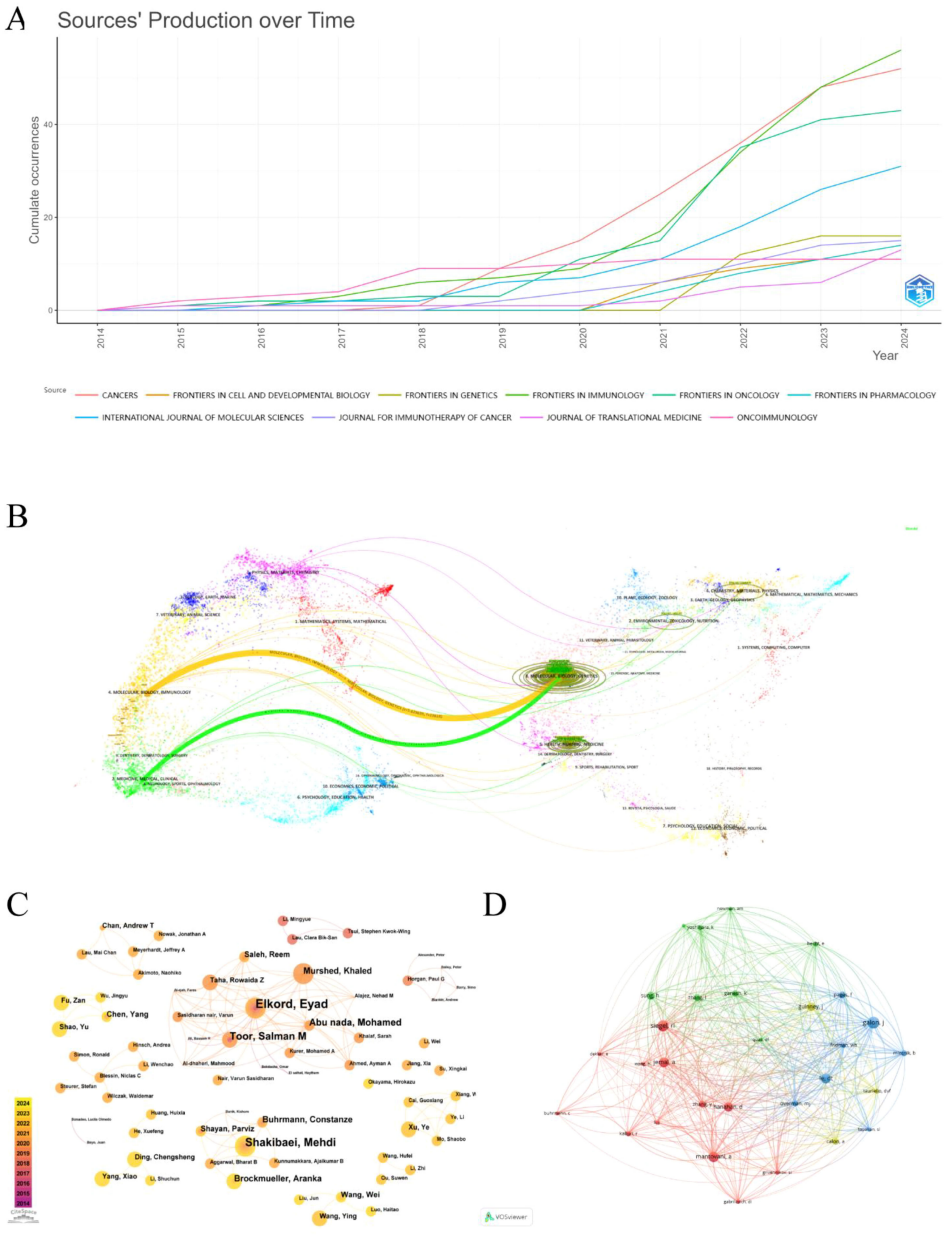
**(A)** Cumulative annual publication charts for journals. **(B)** Journal Dual Map Overlay. **(C)** Author co-occurrence and collaborative network diagram. **(D)** Network diagram of the co-cited authors.

The 748 publications included in this study had been published in 244 different journals. **Supplementary Table S3** lists the top 10 journals by the number of publications in this research area. As it is widely acknowledged that Journal Citation Reports (JCR) and Impact Factor (IF) are significant indicators for evaluating the quality of research and academic impact of journals. We searched the latest JCR partition and IF of the above 10 journals from the Web of Science. Our results suggest that the top 5 journals in terms of publication volume are Frontiers in Immunology (56 articles), Cancers (52 articles), Frontiers in Oncology (43 articles), International Journal of Molecular Sciences (31 articles), and Frontiers in Genetics (16 articles). Of them, the Journal for Immunotherapy of Cancer gets the highest IF (10.3), followed by Oncoimmunology (6.5). Additionally, the H-index, a hybrid quantitative indicator that combines the quantity and quality of scholarly outputs, more accurately reflects the overall impact. Frontiers in Immunology is the journal with the highest H-index and the most citations. (H-index = 20, total citations = 1208). Finally, we utilized the bibliometrix package in R to visualize the annual cumulative publications of the premier 10 journals, as represented in **Figure 3A**. It is obvious that the majority of journals show a notable rise trend, especially Cancers, Frontiers in Immunology, and Frontiers in Oncology, which had explosive growth after 2020 and surpassed other journals during the same period.

The journal dual-map overlay reveals a connection between the citing and cited journals, illustrating the flow of knowledge in the research area in the TME of CRC. On the left and right are the subject areas represented by the citing and cited journals, respectively. Each data point represents a journal, and the colored curves stand for various citation paths (16). The two primary citation paths are depicted in **Figure 3B**; the green curve (z=1.7236, f=7149) and the orange curve (z=5.629635, f=22818) indicate that articles from journals in the field of molecular, biology, genetics are respectively cited by medicine, medical, clinical and molecular, biology, immunology field journals.

### 3.5 Analysis of authors and cited authors

The representative scholars and core research strength of the field can be recognized by analyzing the authors and cited authors. A total of 5509 authors were involved in publishing relevant studies. According to Price’s Law (17), the minimum number of publications by core authors in a certain field is calculated as m=0.749× $\sqrt{n_{max}}$ ≈2.59( $n_{max}$ is the number of publications by the most prolific authors), so authors with three or more publications are defined as core authors in the field, totaling 143 core authors. Listed in **Supplementary Table S4** are the top 10 core authors with the highest number of publications in the field. Among the high-productivity authors, Elkord, Eyad, is the author with the greatest number of publications having published 12 relevant papers in the last decade, followed by Shakibaei, Mehdi (9) and Toor, Salman M (7), which indicates that they are the most popular and innovative researchers in the field. The author collaborations in this domain are visualized via CiteSpace, as pesented in **Figure 3C**. There are 375 nodes and 594 edges, demonstrating that authors with a greater number of publications usually establish great collaborations. In terms of cited authors analysis, 30 authors out of 26,495 cited authors received more than 50 citations, and the close network structure among them is depicted by VOSviewer (**Figure 3D**). The top 10 cited authors and their information are summarized in **Supplementary Table S4**. Galon J is the most frequently cited author (n=168), while Mantovani A from Humanitas University has the highest H-index (H=190). The results above show that both authors have had a significant influence on the research field in the TME of CRC.

### 3.6 Analysis of co-cited and burst references

**Figure 4:**
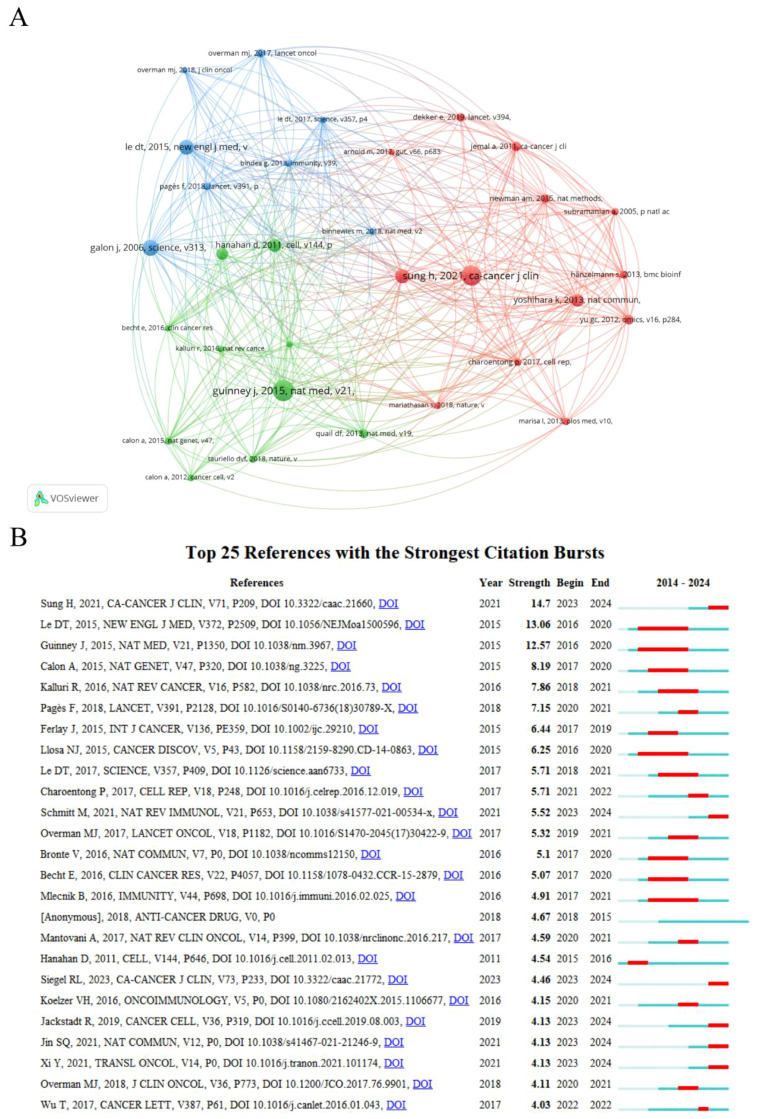
**(A)** Analysis of the co-cited references. **(B)** Top twenty-five references with the strongest citation bursts.

A co-citation connection exists when two or more papers are cited simultaneously by one or more subsequent papers. The analysis of co-cited references can identify core foundational publications, research hotspots within a research area, or connections between different fields (18). A total of 31 out of 36,462 references were co-cited more than 35 times, and the co-citation network mapping was done using VOSviewer, as illustrated in **Figure 4A**. The top 10 references with the most co-citations are listed in **Supplementary Table S5**. These references had all been co-cited at least 50 times and were primarily published in CA: A Cancer Journal for Clinicians and Nature subspecialties between 2006 and 2021. The most co-cited reference was “The consensus molecular subtypes of colorectal cancer” by Guinney, Justin, which was published in Nature Medicine in 2015.

Burst reference analysis is used to identify the publications that are frequently cited by researchers during a specific period, and subsequently to analyze the heated frontiers and trends in the field. CiteSpace was utilized to select the top 25 references with the strongest citation bursts (**Figure 4B**). The red line in the graph represents the duration period of the reference outbursts, starting as early as 2015 and appearing as late as 2023, with durations ranging from 1 to 5 years. Overall, the outbreak strength of these 25 references ranges from 3.94 to 14.61. Among them, the one with the highest outbreak strengths is “Global Cancer Statistics 2020: GLOBOCAN Estimates of Incidence and Mortality Worldwide for 36 Cancers in 185 Countries” (strength=14.61, from 2023 to 2024), published by Sung, Hyuna in CA: A Cancer Journal for Clinicians. The duration of outbreaks in these references with a predicted outbreak year ending in 2024 may be extended due to the incomplete statistics for 2024 data in this study.

### 3.7 Analysis of keywords

**Figure 5:**
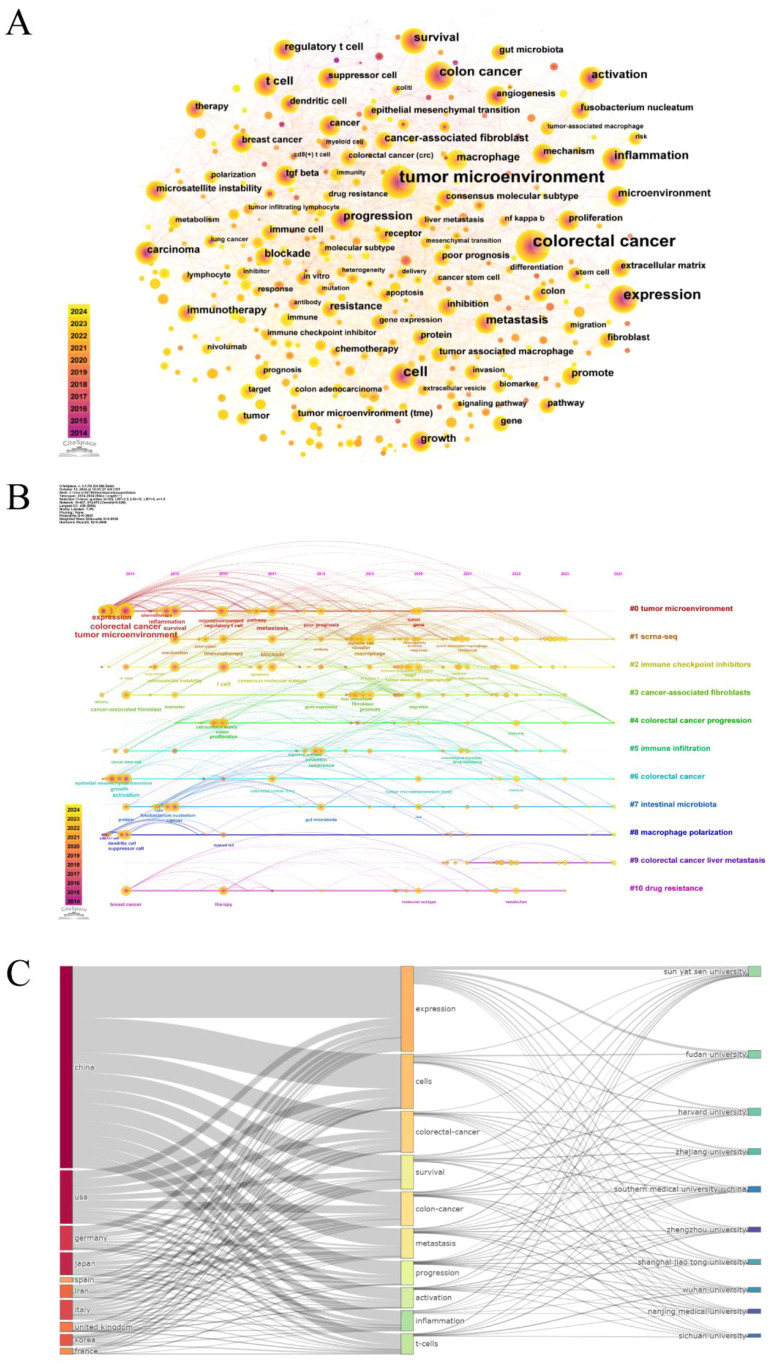
**(A)** Keyword co-occurrence network diagram. **(B)** Timeline diagram of keywords. **(C)** The three-field plot of the tumor microenvironment in colorectal cancer (Left field: countries; Middle field: keywords plus; Right field: institutions).

Keywords are a high-level summary of the core and essence of a paper. Through keyword co-occurrence analysis, we can visually demonstrate the correlation and mutual impact between keywords, as well as uncover the core keywords and potential research hotspots in specific fields (19). As demonstrated in **Figure 5A**, the keyword co-occurrence network consists of 437 nodes and 2479 edges. The size of the nodes signifies the frequency of keyword occurrence, and the higher the frequency, the larger the node. Besides, the color of the nodes represents the year of occurrence, with a darker color indicating an earlier year of keyword occurrence, and the centrality reflects the importance of the keywords. As long as two keywords have appeared in the same article, there is a connecting line between them. It is easy to find that the high-frequency keywords are “tumor microenvironment”, “colorectal cancer”, “expression “, “cell”, “colon cancer”, and so on. **Supplementary Table S6** lists the top 20 keywords and their centrality. The centrality of most keywords is less than 0.1, and the highest centrality is “colon cancer”, which suggests that there are fewer key nodes in the network.

The keyword timeline map combines temporal dimensions and thematic clustering, dynamically reflecting the trend of hotspots in the research field over time (20). The keywords “expression, colorectal cancer, tumor microenvironment, activation” that first emerged in 2014 served as the foundation for this study and continued to be at the forefront of research throughout the study, as displayed in **Figure 5B**. From 2015 to 2019, research themes such as “inflammation, metastasis, microsatellite instability, t cell, therapy” appeared. And the main keywords during the period of 2019 to 2024 were “immune cell, macrophage, liver metastasis, drug resistance, immunity, target”. Ultimately, 11 categories were generated by the log-likelihood ratio (LLR) algorithm: #0 tumor microenvironment; #1 scrna-seq; #2 immune checkpoint inhibitors; #3 cancer-associated fibroblasts; #4 colorectal cancer progression; #5 immune infiltration; #6 colorectal cancer; #7 intestinal microbiota; #8 macrophage polarization; #9 colorectal cancer liver metastasis; #10 drug resistance. Modularity Q primarily evaluates the extent of network structural tightness, with Q=0.3843 (>0.3) implying that the clustering structure is more significant; While Weighted Mean Silhouette S is an indicator applied to assess the homogeneity of the network, S=0.6938 (>0.5) indicates that the clustering is reasonable. What’s more, the three-field plot of countries, institutions, and keywords in the field of colorectal cancer tumor microenvironment was mapped by the bibliometrix package (**Figure 5C**). When it comes to countries, China, the USA, Germany, Japan, Italy, and the United Kingdom contributed a lot to these hotspots, whereas Iran and Korea were less interested in “progression”. France showed little attention to “metastasis, activation”, and Spain is less concerned with “survival, progression”. In the aspect of institutions, Sun Yat-sen University prioritized “expression, survival”, and Wuhan University focused on “cells, t-cells”.

## 4 Discussion

### 4.1 General information

In this study, we searched the publications on the tumor microenvironment of colorectal cancer in the past decade based on the Web of Science Core Collection database and eventually included 748 papers (564 Articles, 184 Reviews) through a series of screening criteria. Subsequently, the bibliometrix package in R, along with CiteSpace and VOSviewer, was applied for bibliometric analysis to reveal the research state, hotspots, and prospective trends of the TME in CRC. The statistical results showed that 748 publications were published in 244 diverse journals by 5509 authors from 1167 institutions in 59 countries, citing 36,462 citations from 3020 journals. Overall, the number of annual publications in the field has consistently increased over the last decade. There was a plateau from 2014 to 2018, a period of inapparent growth in the number of publications, suggesting that it may be in the initial stages of exploration. However, there was a period of rapid growth after 2019, particularly in the last three years, when the number of annual publications exceeded 150. According to the binomial fitting model, a total of 509 publications will be published from 2022 to the end of 2024, accounting for 62.14% of the total. This indicates that more and more scholars are beginning to concentrate on the crucial role of TME in the progression and therapy of CRC, rather than focusing solely on the tumor cells themselves.

In terms of countries and institutions, China and the United States are the major centers of research and collaboration in this area, with both comprising over 60% of the total number of publications. The reason for this is not only that CRC is the fourth and second most common cause of cancer fatalities in China and the United States, but it may also be attributed to the robust research and development infrastructures in both countries and the substantial investment in scientific research and innovation. According to statistics, CRC ranks second in the incidence of new malignant tumors in China, and the group of patients is getting younger and younger (21). This to some extent, has sparked a wave of publications by Chinese researchers and also implies that the field has a good prospect for development. Nevertheless, the average number of citations per publication in China is quite low (15.3), demonstrating a necessity to enhance the quality and academic influence of research. Overall, in terms of the number of national publications globally, CRC and TME-related research has been concentrated in East Asia, Europe and North America, where CRC prevalence and mortality are high. However, if inter-country cooperation is strengthened, certain regions with limited research in this area, such as South America, Southeast Asia, and Africa, could benefit from international collaboration. Therefore, it is important that inter-regional should make efforts to address institutional and cultural differences and to reach a consensus as early as possible in order to ensure successful cooperation. Given that China has the largest overall number of publications, it makes sense that the top 10 institutions are all from China. This highlights the critical role of these institutions in facilitating high academic output, and it is anticipated that these research institutions will make further breakthroughs in the TME of CRC in the coming years. Whereas, we can observe that the majority of the institutional collaborative co-occurrence network is dominated by Chinese institutions, while institutions from other countries are marginal and have low centrality, which manifests the low level of collaboration among international institutions. As a consequence, institutions should transcend regional constraints and augment international cooperation to better promote the development of this domain.

In terms of authors, Elkord, Eyad is the most prolific author in the field, paying attention to the important role of immune cells (such as T-regulatory cells and myeloid-derived suppressor cells) in tumor progression and treatment in the TME of CRC, as well as on immunotherapy and mechanisms of drug resistance in CRC (22–24). To a certain degree, academic journals reflect the research types of publications. Frontiers in Immunology is the journal that publishes the most papers among 244 journals, which means that the research focus may be concentrated on immunology. As shown in **Supplementary Table S3**, only two of the top ten journals belong to the Q2 JCR, while all others are in the Q1 JCR. Nonetheless, as a whole, the impact factors of the journals are all low, confirming that there is still a need to improve the quality of scholarship in the field. The most co-cited paper is “The consensus molecular subtypes of colorectal cancer” published by Guinney, Justin in Nature Medicine in 2015. This research was dedicated to the construction of a classification system of colorectal cancer subtypes based on the expression of consensus genes: CMS1 (microsatellite instability immune, 14%), CMS2 (classical, 37%), CMS3 (metabolic, 13%), and CMS4 mesenchymal, 23%), which informs CRC tumor-targeted therapies across the panorama of CRC tumor microenvironment research (25). Additionally, through the analysis of burst reference, it can be recognized that the research in the TME of CRC has transitioned from the consensus molecular subtypes of colorectal cancer to the study of specific components of the tumor microenvironment, such as the functions of cancer-associated fibroblasts, tumor-associated macrophages in CRC and the mechanisms of intercellular signal transduction.

### 4.2 Research hotspots

Keywords and cluster analysis can obtain the research hotspots and frontiers in the specific field, which will be discussed and summarized in the following parts of this paper.

#### 4.2.1 Major components in the tumor microenvironment of colorectal cancer

Cancer-associated fibroblasts (CAFs) are an important component in the TME of CRC with high heterogeneity and diversity, comprising myofibroblast CAF (myCAF), antigen-presenting CAF (apCAF), inflammatory CAF (iCAF), vascular-associated CAF (vCAF), and ISLRCAF isoforms (26), which play dual roles. Most CAFs are involved in CRC growth metastasis, immune evasion, drug resistance, and ECM remodeling by secreting a series of soluble factors. A study discovered that insulin-like growth factor 2 (IGF2) and insulin-like growth factor 1 (IGF1) secreted by CAFs bind to IGF1-type receptors (IGF1R) on cancer cells, respectively, and activate the downstream Hippo-YAP1 and Akt/mTOR signaling pathways to promote the progression and metastasis of CRC (27, 28). Hiroki et al. conducted experiments to verify that CAFs generated by the proliferation of intestinal crypt peripheral leptin receptor (Lepr) cells shape the tumor-promoting immune microenvironment by expressing melanoma cell adhesion molecule (MCAM) and interacting with interleukin 1 receptor 1, which induces nuclear factor κB-IL34/CCL8 signaling to enhance M2-type macrophage chemotaxis (29). Moreover, CAFs can facilitate CRC immune evasion by recruiting immunosuppressive M2-type macrophages, MDSC, and regulatory T cells (Tregs) or by suppressing the activity of immune cells such as CD8 T cells and NK cells (30–33). Regarding drug resistance, Ren et al. proved that CAFs act as competitive RNA sponges for miR-141 via lncRNAH19 to activate the β-catenin pathway, thereby leading to chemoresistance in CRC (34). Last but not least, YAP is overexpressed in CRC and affects the alignment of key cytoskeletal protein components (F-actin) during the transformation of CAFs, promoting ECM stiffening. In contrast, with increased matrix stiffness, TGF-β motivates increased secretion of activin A by CAFs, which stimulates epithelial cell migration and epithelial-mesenchymal transition (EMT). These findings indicate that inhibiting the TGF-β pathway, activin A, and YAP is a promising target for blocking CRC metastasis (35, 36). On the other hand, certain subtypes like ISLR CAFs exert inhibitory effects on CRC differentiation and metastasis by activating BMP signaling in CRC cells, where specific mediators secreted need to be further investigated (37).

The macrophage, as an integral part of the TME, is a focus of research in this field. It belongs to the category of intrinsic immune cells with a wide range of pathogen-recognizing receptors that can effectively phagocytose and induce inflammatory cytokine production. TAMs are the most abundant immune cell population infiltrated in tumor tissues or present in TME. Macrophage polarization is the process by which macrophages differentiate into two distinct functional phenotypes (M1-type and M2-type) in response to various cytokine stimuli. M1-type macrophages are induced by Th1 cytokines and are responsible for the presentation of tumor-specific antigens and the release of pro-inflammatory cytokines (e.g., IL-1, IL-6, and TNF-α) to augment the anti-tumor immune response. Conversely, M2-type macrophages secrete anti-inflammatory factors (IL-10, TGF-β, VEGF, etc.), which mainly mediate anti-inflammatory responses and Th2-type immune responses, thus participating in angiogenesis, EMT, immunosuppression, and tumor metastasis (38, 39). It is worth noting that there is no absolute antagonistic distinction between the M1 and M2 phenotypes, and macrophage polarization is a dynamic process in which their ratios change in response to the microenvironment. This provides an opportunity for the targeted reprogramming of TAMs for drug development. Jing et al. by conducting vitro and vivo experiments, discovered that Hydroxygenkwanin (HGK) activates a variety of signaling pathways to help the polarization of TAMs towards the M1-type macrophages and inhibit their polarization to M2-type macrophages to obstruct CRC peritoneal metastasis (40).

The human intestinal microbiota constitutes a complex ecosystem with thousands of microorganisms, including bacteria, fungi, viruses, archaea, and parasites (41). Notably, CRC is distinguished from many other cancers due to its close connection with the gut microbiota. In recent years, an increasing number of studies have proved that the gut microbiota and its metabolites affect the immune microenvironment of CRC leading to tumor progression, metastasis, and response to therapy, especially bacteria of the genus Clostridium. Hu’s research pointed out that Fusobacterium nucleatum (Fn) regulates the ratio of M1 to M2 macrophages through the activation of TLR4/NF-κb/s100A9 signaling, and promotes M2-type macrophage polarization, which contributes to TME re-editing and stimulates CRC metastasis (42). Sakamoto et al. found that Fn is associated with a lower density of CD8+ T cells and a higher density of MDSC in CRC liver metastasis (43). Furthermore, Jiang et al. observed that succinic acid produced by Fn reduces the number of CD8+ T cells in TME by blocking the cGAS-interferon-β pathway, which results in immunotherapy resistance (44). What’s more, the role of some non-bacterial gut microbiota such as viruses and fungi in the TME of CRC requires further investigation.

#### 4.2.2 Association between colorectal cancer therapy and TME

As shown by the keywords and cluster analysis, the link between colorectal cancer therapy and TME is one of the hot topics, particularly in the research of immunotherapy and drug resistance targeting TME. Immune checkpoint inhibitors (ICIs), a crucial category of immunotherapy, have been proven to make substantial advancements in the treatment of colorectal cancer (CRC). It is designed to block immunosuppressive tumor signaling by targeting receptor or ligand checkpoint proteins, with typical targets including programmed death-1 (PD-1), programmed death-ligand 1 (PD-L1), and cytotoxic T lymphocyte-associated antigen-4 (CTLA-4) (45). PD-1/PD-L1 inhibitors have been shown to exert antitumor effects by decreasing the depletion of CD8+ cytotoxic T cells and increasing related chemokines and cytokines in TME (46, 47). Therefore, due to the presence of tumor microenvironment feature dependency in clinical practice, ICIs are more effective in patients with deficient DNA mismatch repair/high microsatellite instability (dMMR/MSI-H) CRC, which has a high tumor mutation burden and strong tumor immunogenicity. In contrast, patients with proficient DNA mismatch repair/microsatellite stability (pMMR/MSS) CRC have poorer efficacy. As early as 2015, a phase II clinical trial (KEYNOTE-016) reported that the immune-related objective remission rate and immune-related progression-free survival rate of pembrolizumab (PD-1 inhibitor) in patients with dMMR-type refractory progressive mCRC were 40% and 78%, respectively, compared with 0% and 11% in pMMR-type CRC (48). Since then, ICIs have crossed into an era of rapid clinical translation, and the FDA approved pembrolizumab for the first-line therapy of dMMR/MSI-H mCRC patients in 2020 (49). Subsequently, clinical studies about nivolumab, dostarlimab, and ipilimumab have progressively been carried out. With further research on TME, new immune checkpoints have been continuously discovered, such as lymphocyte activation gene 3 (LAG-3), V structural domain Ig suppressor factor of T-cell activation (VISTA), T-cell immunoglobulin, and ITIM structural domain (TIGIT) (50).

Although ICIs have achieved successful clinical translation in dMMR/MSI-H CRC, 80% to 90% of the clinical patients are pMMR/MSS CRC patients, which means that the application of ICIs is severely restricted. One of the reasons may be the lack of cytotoxic T-lymphocytes (CTLs) infiltration in the tumor microenvironment of pMMR/MSS-type colorectal cancers, along with low expression or absence of MHCI and MHCII molecules, resulting in immune escape, which is commonly recognized as “cold tumors” (51, 52). More importantly, the TME of CRC can induce immune resistance through other multiple mechanisms, including the infiltration of immunosuppressive cells (Tregs, MDSC, TAMs), oncogenic signaling pathway-mediated immunosuppression (WNT/β-catenin, TGF-β, interferon-γ), tumor metabolism (lactic acid accumulation, hypoxia, glycolysis), overexpression of other suppressive immune checkpoints and molecules (LAG-3, TIM-3, IGIT), and gut microbiota (53–55). In particular, TME also leads to targeted drug resistance. Dennis et al. found that targeting vascular endothelial growth factor receptor (VEGFR) drugs markedly reduced the infiltration of TAMs and Tregs into the tumor microenvironment to inhibit immune escape (56). Currently, combination therapies are mainly used in the clinic to improve immune efficacy, for example, ICIs combined with chemotherapy, radiotherapy, or targeted therapy, ICI two-drug combination, and neoadjuvant immunotherapy.

In summary, ICIs enhance anti-tumor effects by blocking immune checkpoint molecules, reactivating the immune response effect of T-cells against tumors, and restoring the immune microenvironment of tumors. Compared with traditional therapies, ICIs directly target the immunosuppressive pathway and solve the problem of immune escape. At the same time, it enhances the efficacy of dMMR/MSI-H type CRC, and overcomes the immunosuppressive tumor microenvironment of pMMR/MSS type CRC patients by combining treatment with radiotherapy or targeted drugs to enhance the efficacy and delay the occurrence of drug resistance. Furthermore, by combining ICIs treatment with biomarker (e.g., PD-1 expression, TMB, TIL count) detection, precision medicine is realized to help screen patients suitable for ICIs treatment. In addition, ICIs are expected to significantly improve the survival of patients with advanced or metastatic CRC by stimulating the persistent activity of immune memory cells. In the future, researchers need to develop more effective biomarkers to screen the beneficiary population, explore novel ICIs and combination therapies to overcome drug resistance, and study the CRC tumor immune microenvironment in depth to guide individualized treatment.

#### 4.2.3 Tumor microenvironment mediated liver metastasis in colorectal cancer

The liver is the most common site of CRC metastasis. Statistics reveal that over 20% of CRC patients present with liver metastasis at the time of initial diagnosis, and more than 50% of primary CRC will develop liver metastasis (57, 58), which comes along with a dismal prognosis. Therefore, understanding the mechanism of colorectal cancer liver metastasis (CRLM) will be beneficial in finding precise therapeutic targets. Cancer stem cells, epithelial-mesenchymal transition, and tumor microenvironment are considered to be the three classical changes in CRC that promote liver metastasis, and this process usually interacts and overlaps (59). Normal colorectal cells undergo oncogenic mutations in various genes like BRAF, TP53, and KRAS to form aggressive tumor stem cells (CSC), which are then implanted into the liver tissue through EMT, ECM remodeling, and peripheral tissue vascular migration (60–62). Secondly, the tumor microenvironment of colorectal cancer is a necessary prerequisite for its development and metastasis, and a variety of immune cells, cytokines, chemokines, exosomes, metabolic reprogramming, and interactions with cancer cells collectively facilitate CRLM (63). Thirdly, pre-metastatic ecological niches in the liver immune microenvironment play a key role in promoting CRLM, especially liver sinusoidal endothelial cells (LSEC), Kupffer cells (KC), and hepatic stellate cells (HSC) (64). Consequently, immunotherapy targeting the tumor microenvironment like chimeric antigen receptor T-cell therapy (CAR-T) and tumor vaccines will be the future research hotspots for CRC precision therapy.

#### 4.2.4 Research methods in the field of the TME in CRC

Single-cell RNA sequencing (scRNA-seq) is a high-throughput experimental technique to quantify the gene expression profile of a specific cell population by analyzing the RNA transcripts of each cell individually. Liang et al. proved, using scRNA-seq and *in vitro* experiments, that CRC cell-derived exosome miR-106a-5p induces the polarization of M2 macrophages through the down-regulation of SOCS6 and activation of the JAK2/STAT3 signaling pathway. Finally, these M2 macrophages reciprocally enhance CRC liver metastasis (65). Li et al. discovered by scRNA-seq combined with a prospective cohort study that d-MMR/MSI-H CRC patients who achieved pathological complete remission (pCR) post-PD-1 inhibitor treatment had a decrease in CCL2 fibroblasts, while CD20 B cells and HLA-DRA endothelial cells increased (66).

## 5 Limitations

It is undeniable that this study has several limitations. At first, our data were only obtained from the WoSCC database and only English publications in the form of Articles and Reviews were included, which may have caused incomplete data and analysis results, resulting in literature database bias. Moreover, there are still a number of important publications in other languages that were not included in this study, missing the potential for different perspectives and insights from other languages, which may have an impact on the overall understanding of global research changes, and consequently lead to language bias. Nonetheless, due to the very small percentage of non-English articles and the high coverage of WoSCC in the vast majority of studies. Therefore, the trend of our study remains an important reference. Secondly, our search finished on September 6, 2024, perhaps overlooking some recently updated papers during the completion of the study. Also, some high-quality studies published recently may have been disregarded because of low citation counts. Thirdly, the search results may not be the same owing to the different range of databases purchased by the organizations. Fourthly, there may also be minor discrepancies in some of the analysis results since different software versions and analysis methods.

## 6 Conclusions

In conclusion, we used bibliometric methods to analyze, visualize, and summarize the publications on tumor microenvironments of colorectal cancer from 2014 to 2024 in this research. At present, the field is in a rapid development stage, with significant contributions from China and the United States. Future research in this area needs to overcome the low average citation rate of Chinese research and promote international cooperation. First, Chinese scholars should improve their own research quality and innovation. Second, Chinese scholars should strengthen academic exchanges and international cooperation, improve the research evaluation system to incentivize scholars to publish high-quality papers, and strengthen research team building. Third, it is important to break down institutional and cultural differences and reach a consensus as soon as possible to ensure the success of cooperation. In addition, the main components and signaling in the tumor microenvironment of colorectal cancer, the association between tumor microenvironment and colorectal cancer treatment, and liver metastasis of colorectal cancer will become the research hotspots in this field.

## Data Availability

The original contributions presented in the study are included in the article/**Supplementary Material**. Further inquiries can be directed to the corresponding author.
